# COVID-19 lockdown disrupts support networks integral to maintaining foot health: a mixed-methods study

**DOI:** 10.1186/s13047-021-00486-4

**Published:** 2021-06-30

**Authors:** Lindsey Cherry, Lucy Gates, David Culliford, Karen Walker-Bone, Mari Carmen Portillo

**Affiliations:** 1 School of Health Sciences, Faculty of Environmental and Life Sciences, University of Southampton, Building 67, University Road, Southampton, SO17 1BJ UK; 2 Department of Podiatry, Solent NHS Trust, St Mary’s Community Hospital Campus, Portsmouth, PO3 6DW UK; 3 Versus Arthritis Centre for Sport, Exercise and Osteoarthritis, University of Southampton, University Road, Southampton, SO17 1BJ UK; 4 NIHR Applied Research Collaboration for Wessex, University of Southampton, University Road, Southampton, SO17 1BJ UK; 5 MRC Versus Arthritis Centre for Musculoskeletal Health and Work, Southampton General Hospital, Southampton, SO16 6YD UK

**Keywords:** Foot heath, COVID-19, Pain, Physical activity, Exercise, Infection, Support, Self-care, Self-management, Unmet need, Survey, Mixed methods

## Abstract

**Background:**

In response to the COVID-19 pandemic, populations were advised to remain at home to control viral spread. Government-mandated restrictions on free movement affected individuals’ engagement with physical activity, with reported increases leading to biopsychosocial health benefits and conversely increased sedentary behaviour leading to poorer health. Good foot health is key to enabling physical activity and maximal participation in activities of occupation and daily living.

**Methods:**

A population-based cross-sectional study was performed, using a web-based platform. Quantitative and qualitative data were captured through responses to closed and open survey questions. Anybody with a foot health condition was eligible to participate in the online survey. Links were sent through professional networks, support groups and charities, using a snowball strategy to maximise participation.

**Results:**

Two hundred fifty-five respondents completed the survey. Most (*n* = 193, 75.69%) reported an ongoing foot pain or problem that had been present for 4 weeks or longer, whilst 49 respondents (19.22%) noted a new pain or problem. Pain was the most frequently reported symptom (*n* = 139, 54.51%), whilst change in appearance of the foot was also commonly reported (*n* = 122, 47.84%), often alongside the observable presence of swelling. Musculoskeletal foot symptoms were frequently reported (*n* = 123, 48%), and were significantly associated with reported reduced physical activity (X^2^ = 6.61, *p* = 0.010). Following qualitative analysis five themes and 11 subthemes emerged, informed by 49 independent codes. A central theme of lockdown disrupting support networks, both formal (healthcare providers) and informal (friends or family members) emerged. The 5 sub-themes were: 1. foot pain is a constant companion, 2. self-care, 3. ‘cope or crumble’ scenarios, 4. future intent to access healthcare and 5. reduced ability to undertake physical activity.

**Conclusions:**

Pain was the most frequently reported foot problem during COVID-19 lockdown restriction. Lockdown restrictions disrupted support networks integral to maintaining foot health. Poor foot health impacted people’s ability to remain physically active. Complaints previously considered relatively ‘minor’ such as support for skin and nail care, were found to be exacerbated by restricted support networks, leading to greater negative impact.

**Supplementary Information:**

The online version contains supplementary material available at 10.1186/s13047-021-00486-4.

## Background and rationale

In response to the global severe acute respiratory syndrome coronavirus (SARS-COV2, also termed COVID-19) pandemic, whole nations have been advised to remain at home in attempts to control viral spread and limit critical overburdening of healthcare systems [1, 2]. The related socioeconomic impact of the virus is increasingly widely reported, as are the significant primary and secondary health implications [1, 3–5]. Government-mandated restrictions to movement (termed ‘lockdown’), meant that the amount or type of physical activity with which people engage has changed [2, 6], with some individuals reporting increases leading to biopsychosocial health benefits and conversely others reporting increased sedentary behaviour leading to poorer health [2, 5, 7, 8]. Various reports have highlighted that the impact upon overall mobility has been significant [5, 7] and has disproportionately affected regions with greater social and health inequality [1]. Individuals with pre-existing disease affecting mobility have been significantly impacted [4], as have health and care systems [9, 10].

Good foot health is key to enabling people to remain physically active and mobile, and able to participate fully in activities of occupation and daily living [11]. With the emergence of a recently recognised post-COVID-19 syndrome causing persistent symptoms, fatigue, dyspnoea, pain and psychological effects after COVID-19 infection, rehabilitation is likely to include weight-bearing activity [12], and as such attention to foot health is a central rehabilitative concern. Being able to walk and move around freely is a cornerstone of healthy living and could significantly confound rehabilitation potential for post-COVID-19 disease. Indeed, Menz [13] highlighted the particular importance of foot health in the context of overall wellbeing for the older adult in a recent review, and there is a wealth of literature highlight the importance of early and continued foot health monitoring for people living with pre-existing long term conditions such as Diabetes Mellitus or chronic limb-threatening ischemia [14–17].

There are currently no data reporting the impact of the global COVID-19 pandemic, and related lockdown restrictions, on foot health [11], however Rogers et al. acknowledge the important role that Podiatrists can play in maintaining foot health and possibly preventing overburdening of healthcare systems [18]. Knowledge about this will enable better-informed planning and provision of healthcare and a wider public health knowledge about the effects of lockdown on physical exercise and to what extent these effects were driven by foot health. Further, such information could be used to develop timely and targeted intervention, that could be provided in accordance with lockdown restriction, e.g., self-care guidance or inclusion of foot health within rehabilitation programmes or public health messaging [12]. Thus, the main aim of this research was to determine the type of foot health problems experienced by people during the COVID-19 pandemic and explore their impact.

## Methods

### Ethical approval and consent

Ethical permission to undertake this work was granted by the University of Southampton, Faculty of Environmental and Life Sciences, UK, research ethics committee (ref: 56806).

Potential respondents were able to view the participant information on opening the survey weblink, where they also found written evidence of ethical approval. Having read this information, respondents confirmed their informed consent by clicking the ‘next’ button. Parents were able to answer for their child, however the person completing the survey confirmed by clicking the next button that they were aged 18 years or over. Respondents were able to discontinue survey completion at any time and only submitted complete survey responses were analysed.

### Design, aim and context

This was a concurrent mixed-methods study [19], with priority on the quantitative methods. The cross-sectional survey was web-based using ‘Survey Monkey’®. Quantitative and qualitative data captured in the form of open survey questions, were collected concurrently to address the primary research question ‘what challenges to foot health are people experiencing during the COVID-19 pandemic?’. The research objectives were to 1. determine the comparative number and nature of foot health symptoms, and 2. develop understanding of potential relationships between self-reported foot health complaints and the impact of COVID-19 lockdown.

A mixed-methods approach was chosen to enable an analysis of free text responses, such that reasonable understanding of the unique context of respondents related to their changing circumstance amidst the pandemic could be explored. The qualitative responses were used to provide context and meaning to the quantitative data captured. An inductive exploratory approach was applied via a thematic analysis framework to qualitatively explore meaning and context. Overall, a pragmatic research paradigm was used to bring together a positivist quantitative and qualitative approach to this research.

The research was undertaken 1st June 2020 - 15th July 2020 during a global pandemic whilst many governments had imposed ‘lockdown’ restriction upon their citizens, meaning a restriction in personal freedoms and ability to leave their place of residence for any reason not considered as legitimate within the individuals geographical region. The researchers were subject to the same restrictions as those they were researching at the time of conducting this project. All researchers remained active in their roles during the restriction, with LC and KWB continuing to practice clinically within the UK.

### Participants and sampling

The survey was intended for completion by any person with foot symptoms. Parents were able to answer for their child/young adult. No geographical restrictions were applied, and respondents indicated their Country of residence at the time of survey completion. Participation was not possible if the individual was unable to read or write in English language; unable to provide informed consent, unable to access SurveyMonkey with a suitably enabled device. People not experiencing foot health symptoms at the time of survey were ineligible.

The survey was initially distributed across academic, professional, and social networks known to the research team, including academic partners (e.g., inclusion within a UK University wide newsletter), email to footwear retailers, charitable organisations, voluntary organisations, formal professional networks with a publicised single point of contact, and via social media networks such as Twitter®. A snowball sampling approach was encouraged, whereby the researchers actively encouraged further spread and distribution of the survey by recipients. The survey remained opened for 45 days after initial launch.

### Survey development and conduct

The survey content was co-designed by members of the research team with Podiatry clinicians (*n* = 5) from Solent NHS Trust, UK, in an iterative design and face validity checking process, until consensus on a final draft was achieved. The survey included 22 closed and 9 open questions about foot health and care, and physical activity.

The survey was entered into a virtual framework for online distribution using SurveyMonkeyPro® (2020) to allow secure anonymous survey participation. A web-based link to the online survey was distributed as above.

Data was held within SurveyMonkey and extracted as an Excel file (Microsoft 365®, 2020) for analysis.

### Analysis

All quantitative analysis was completed in Stata version 13.0 (Stata Corp, College Station, Texas, USA). Prior to analysis, data distributions were checked for inconsistencies, outliers, and missing information.

Descriptive data for demographic characteristics were calculated and presented as means and standard deviations or frequencies and percentages. Data concerning the type of foot pain or problem reported were categorised from free text responses into groups corresponding to the primary physiological system affected: musculoskeletal, integumentary, nervous, circulatory, immune, endocrine, digestive, reproductive, respiratory, or renal, and pain and footwear. Where multiple systems were affected, both categories were included within analyses.

Chi-square tests of association were performed to examine the relationship between foot pain and musculoskeletal foot problems, and the relationship between foot pain and integumentary related foot problems. Predictors for the dichotomised foot pain outcome were also assessed using logistic regression models to obtain estimates of odds ratios and 95% confidence intervals (95% CIs). The following covariates were used in the adjusted models for foot pain: age, footwear, and the following foot related problems: circulatory, integumentary, musculoskeletal, endocrine, immune, neurological.

For the qualitative component of survey analysis (open questions), a thematic analysis approach was used to explore the content of question responses, identify patterns within data, and describe and interpret their meaning and importance. A summary of the phases of qualitative thematic analysis is illustrated in Table 1, adapted from a proposed analysis framework by Nowel et al [20].

**Table 1:** Summary of integrated quantitative and qualitative analysis

Phase of thematic analysis	Means of establishing trustworthiness
Phase 1: Data familiarisation	Prolonged engagement with the data including completion of initial quantitative analyses (LC, LG & DJC)
	Document and discuss theoretical and reflective thoughts (all researchers)
Phase 2: Code generation	Document thoughts about potential codes (LC & LG independently)
	Researcher triangulation (LC, LG & MC)
	Log audit trail of code generation (LC & LG)
Phase 3: Theme generation	Theme generation (LC and LG independently)
	Research triangulation (LC, LG & MC)
	Document theme connections (LC & LG)
Phase 4: Theme confirmation	Research triangulation (LC, LG & MC)
	Diagramming to make sense of theme connections (LC & LG)
	Test for referential adequacy by returning to raw data and completing quantitative analyses (LC & LG)
	Team consensus on themes (all researchers)
	Documentation of theme framing process
Phase 5: Integration with quantitative data	Integration of quantitative data within qualitative context and cross-referencing for credibility (LC & LG)
	Researcher triangulation (LC, LG & MC)
Phase 6: Report production	Generate full data report for dissemination

### Integration process

The following process was followed to integrate qualitative and quantitative analyses and findings in this study.

Steps 1 and 2. After completing the initial descriptive quantitative analysis, these findings provided the framework for qualitative evaluation, as per a mixed methodological approach set out by Creswell et al. [19]. Consequently, first-order codes were developed and selected independently by two researchers (LC and LG), and subsequently triangulated. These codes were discussed and harmonised in the sense of merging findings from the qualitative and quantitative data especially to validate and understand the quantitative survey findings [19]. This initial coding was validated with a third independent referee, MCP.

In step 3, second order codes, building conceptual connections between first order codes were created to illustrate and unify themes and subthemes and synthesize the qualitative findings.

Step 4 included the refinement of the quantitative analysis, which was used to inform the qualitative diagraming process and referred to for referential adequacy.

Finally, in step 5, findings of both quantitative and qualitative analyses were combined in the first report draft and key quotes identified. Cross-referencing between all three researchers took place throughout steps one to five, to enhance the trustworthiness of the research findings. An auditable log of thematic decision making was maintained within which versions of thematic refinement, leading to the integrated theoretical framework, were stored.

## Results

A total of 255 respondents completed the survey, two of whom indicated that they were completing the survey on behalf of their child aged under 18 years. Most respondents (*n* = 249) were from the United Kingdom, although responses were also received from Oman (*n* = 3), Spain (*n* = 1), Guyana (*n* = 1), and Australia (*n* = 1). A summary of respondent demographic characteristics is shown in Table 2.

**Table 2:** Survey respondent demographic information (*n* = 255)

Characteristic		Group value
Age, mean (SD) *n* = 10 (3.92%) missing		57.77 (13.0)
Gender, n (%) female *n* = 3 (1.18%) missing		216 (84.7)
Ethnicity, n (%) *n* = 3 (1.18%) missing	British	209 (82.0)
	Irish	4 (1.6)
	African	2 (0.8)
	Asian	3 (1.2)
	Other mixed background	11 (4.3)
	Other white background	21 (8.2)
	Other ethnic group	2 (0.8)
Residing area, n (%) *n* = 1 (0.39%) missing	Rural	17 (6.7)
	Semi-rural	115 (45.1)
	Urban	122 (47.8)
Shielding status, n (%) *n* = 1 (0.39%) missing	At home shielding	73 (28.6)
	Mostly at home isolating	153 (60.0)
	Regularly outside but mostly sedentary	21 (8.2)
	Regularly outside and physically active	7 (2.8)

Most respondents (*n* = 193, 75.7%) reported foot symptoms which had been present for 4 weeks or longer, but 49 respondents (19.2%) noted a new problem. The duration was missing for 13 (5.1%) respondents.

Pain was the most frequently reported symptom, (*n* = 139 (54.5%)). Change in appearance to the foot was also commonly reported (*n* = 122, 47.8%), often alongside reporting the presence of swelling. Most foot problems were musculoskeletal (*n* = 123 (48%). A summary of response frequencies for other categories is shown in Table 3. Additionally, *n* = 8 (3.1%) respondents reported having had an infection which required antibiotic prescription.

**Table 3:** A summary of self-reported foot problems provided from free text responses

Foot problem	Present n (%) ^**a**^
Musculoskeletal	123 (48.2%)
Integumentary	61 (23.9%)
Nervous	17 (6.7%)
Circulatory	5 (2.0%)
Immune	6 (2.4%)
Endocrine	1 (0.4%)
Digestive/ reproductive/ respiratory/ renal	0 (0%)
Pain	139 (54.5%)
Footwear	11 (4.3%)

^a^missing data from *n* = 13 (5.1%) respondents

### Emergence of qualitative themes and an explanatory framework demonstrating the impact of COVID-19 upon foot health

**Fig. 1:**
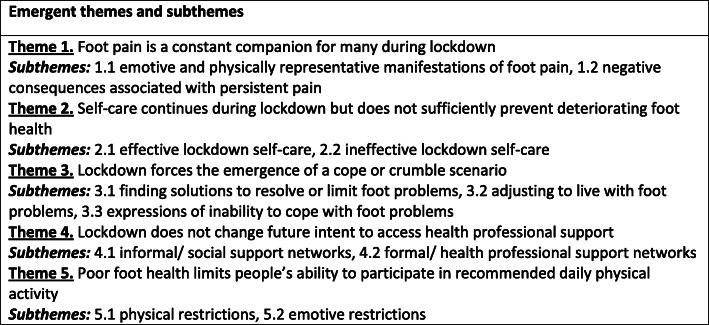
Themes and subthemes emerging from integrative analysis

A total of five themes and 11 subthemes were identified (Fig. 1). The full thematic analysis summary, including itemisation of the 49 derived codes and representative quotations, is available in appendix one.

**Fig. 2:**
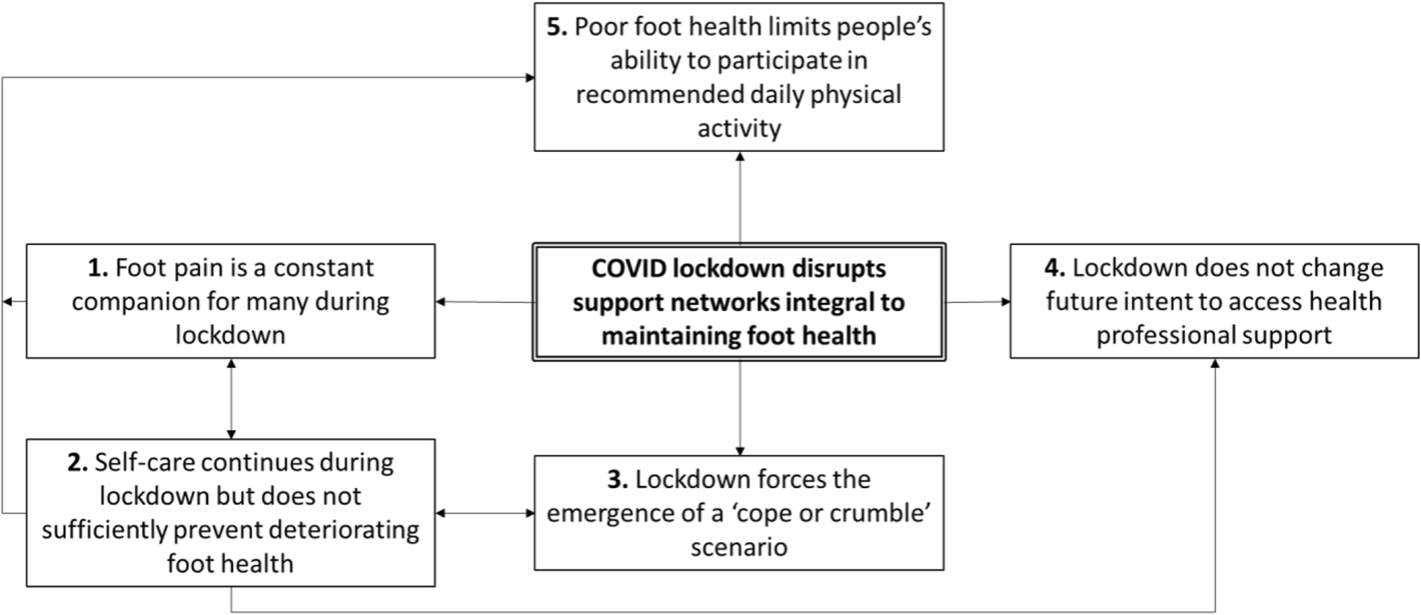
An explanatory framework demonstrating the impact of COVID-19 upon foot health

Integrative analysis identified one over-riding explanatory contextual theme, that of lockdown as a disruptor to support networks integral to maintaining foot health. Using an integrated analysis approach, an explanatory framework demonstrating the impact of COVID-19 upon foot health was developed (Fig. 2).

#### Theme 1. Foot pain is a constant companion for many during lockdown

Fifty-five percent of participants reported foot pain (*n* = 139). Pain was more common amongst those reporting musculoskeletal foot conditions than others (X^2^ = 12.00, *p* = 0.001). Foot pain was less common amongst those who did not report an integumentary condition (X^2^ = 10.61, *p* = 0.001) (Table 4). The specific pathologies relating to foot pain were largely unclear from survey responses. However, the impact of foot pain was highly evident within the qualitative text and there was a sense that those with MSK complaints accepted foot pain as inherently part of the condition.

**Table 4:** Cross tabulations of those who did and did not report foot pain versus those who i) did and did not report a foot problem related to musculoskeletal system and ii) those who did and did not report a foot condition related to the integumentary system

	Not reporting foot pain, n (%)	Reporting foot pain, n (%)
Not reporting MSK problem, n (%)	38 (31.7)	82 (68.3)
Reporting MSK problem, n (%)	66 (53.7)	57 (46.3)
Not reporting integumentary problem, n (%)	67 (36.8)	115 (63.2)
Reporting integumentary problem, n (%)	37 (60.7)	24 (39.3)

Qualitative findings also illustrated associations between experience of pain and physical signs of poor foot health, such as swelling or deformity, suggesting a potential belief that pain is physiologically driven:

*“even though I had surgery on all my toes for bunion joint and dropped joints, this was some years ago and I am now suffering with pain in some toes and difficulty walking as 2 little toes on right foot turn right under, so they get sore when walked on” (participant #5).*

There were numerous references made by respondents to the negative consequences associated with persistent pain and observation of how COVID lockdown exacerbated such beliefs.

*“I have had foot pain for over a year caused by an injury (likely stress fracture/ligament damage) that causes aggravation to mid-foot during exercise/walking. Too much aggravation leads to me not being able to walk on that foot without limping. I have not been able to do much exercise since working from home and losing out on the movement throughout the day seems to have made things worse” (participant #78).*

In addition to foot problems limiting activity during COVID lockdown, respondents also noted a range of negative emotions associated with foot pain, including fear of movement, reduced motivation to exercise, and finding walking and mobility harder.

*“I find I’m less motivated to go out for walks as not sure how much the pain may flare up and then I find it difficult and painful to walk home. If I do go out, I try to use the car or make sure it’s short distances from home”; “Pain is making me reluctant to move about and no reason to push myself because of the shielding” (participant #85).*

The lack of routine and a structured day associated with COVID lockdown also appeared to impact typical pain coping mechanisms and in particular sleep patterns:

*“The pain causes … I am sleeping a lot in the daytime but up in the night, sitting in my recliner chair for 1-2 hours, usually around 4am” (participant #177).*

Similarly, one respondent also noted the potential link between a change in lockdown diet, weight gain and foot pain:

*“I have also put-on weight through lack of exercise (and easy access to unsuitable food!) but feel the excess weight may have contributed to my foot pain” (participant #176).*

Overall, perceived ability to adapt and cope with the complexity of pain appeared limited amongst respondents, whilst the impact of pain was moderate to severe.

#### Theme 2. Self-care continues during lockdown but does not sufficiently prevent deteriorating foot health

During the COVID lockdown 192 (78.05%) respondents reported attempting to undertake self-care to support their foot health. The proportion of those undertaking self-care did not differ between those with long-term (over 4 weeks) and those with a new (within 4 weeks) foot problem (X^2^ = 0.03, *p* = 0.856), (Table 5).

**Table 5:** Cross tabulation of those undertaking self-care comparing those with a new problem and those with longstanding problems

	Ongoing pain	New pain	Total
Self-caring for feet	39 (20.5%)	10 (21.7%)	49 (20.8%)
Not self-caring for feet	151 (79.5%)	36 (78.3%)	187 (79.2%)
**Total**	190 (100%)	46 (100%)	236 (100%)

Qualitative data analysis indicated that self-care was a frequently referenced activity of great importance to respondents. There was a particular emphasis on undertaking skin or nail self-care and in some cases, this extended to the treatment of nail or skin infection:

*“I remove as much fungus as I can with a nail-file or small knife”; “I wear wound covers on my blisters to stop infection” (participant #91).*

Strategies to support self-care described as effective included use of over-the-counter products, massage, use of hot/cold therapy, rest and/or elevation of the foot, use of footwear and insoles, exercise, and use of medications:

*“Swelling makes skin more red and very dry. Difficult to keep feet clean as can’t reach them. Bought a gadget to try to apply moisturiser” (participant #149); “occasionally when it’s bad will get a cold bottle from the freezer to roll under my feet” (participant #85); “Raising leg as often as possible (during working hours not so easy as sat at dining room table to work” (participant #87).*

In contrast, several respondents also reported detrimental impact of ineffective skin or nail self-care, and these observations were typically in relation to inability to manage callus or nail growth, or uncertainly around product use, in particular when they usually relied upon professional advice or treatment:

*“get a lot of build-up of callus under my metatarsals. This is usually removed every 6 weeks but, because of lockdown, is building up (in spite of my trying to get rid of it)” (participant #125);*

The contrast in views about effectiveness of self-care appeared to be concordant with the contrast in overall emotive expression of either stoicism or helplessness that is amplified by experiences in lockdown, as discussed in theme three. Here, the overall theme of disruption to support networks became evident. Forty-four (17.81%) respondents were receiving some form of help or support for their foot health during the lockdown period, 30 of whom (68.18%) reported this support was meeting their needs. However, 204 (84.3%) respondents reported an intention to seek external help or support for their foot health once the lockdown period ended and they were able to do so, suggesting that whilst self-care was prevalent, it was not sufficiently addressing health needs.

#### Theme 3. Lockdown restrictions force the emergence of a cope or crumble scenario

A typically polarised response to the challenge to living with poor foot health during a period of COVID lockdown emerged and we observed that comment relating to this theme was highly prevalent within the free-text responses, and therefore considered it worthy of inclusion as a separate theme. Finding personalised solutions and adjustment to daily routines or activities appeared to provide the greatest sense of coping and stoicism, largely in the context of self-management. The support networks underpinning a person’s ability to find solutions or adjustments to cope with poor foot health appear to be intricately linked to the emergence of a cope or crumble scenario. The strong sense of stoicism described appeared to be driven by a wish to prioritise physical activity above pain avoidance, or the potential to cause more physical harm to the foot:

*“I do try to walk even with the pain but the pain beats me in the end” (participant #165); “I push through the pain and limp if need be” (participant #166).*

Conversely, whilst some respondents described adjustments to daily living, for many others there were notable expressions of concern or inability to cope:

*“I worry about the long-term effect of my bones getting so out of alignment. I am normally an active person” (participant #88).*

#### Theme 4. Lockdown restrictions do not change future intent to access health professionals

A reliance upon informal support networks was reported by many respondents. However, several participants also noted their reliance upon formalised support networks and healthcare professionals, reporting their intention to access support as soon as it becomes available again:

“*… under current circumstances trying to look after feet is extremely difficult due to medical conditions … have to wait until major problems before can be seen which will probably mean more visits – not cost effective and potentially not safe for me to attend and means more time for staff” (participant #24);*

There were no examples of respondents finding new self-care strategies, relating to theme three, support networks or solutions that meant that they no longer intended to access healthcare professionals in the future. It would appear the lockdown restrictions did not drive innovation or change behaviour relating to self-care suggesting that professional input remains an important facilitator of self-care and there is intent to seek this support once access restrictions are removed. However, it should be noted that this topic was not explicitly questioned within the survey, and thus this observation could reflect the lack of direct questioning within the survey design.

#### Theme 5. Poor foot health limits people’s ability to participate in recommended daily physical activity

Important emphasis was placed upon participation in daily activity, both in continuing public health messages of the time and in mainstream media. However, 151 (59.2%) of respondents reported a reduction in physical activity, with 57 (22.4%) reporting similar amount and 46 (18.0%) reporting increased activity (*n* = 1 missing). Notably, 128 (50.2%) respondents cited their poor foot health as affecting their ability to remain active during the COVID-19 lockdown (*n* = 5 missing). Quantitative analysis revealed a significant relationship between (a) pain and (b) musculoskeletal foot conditions and reduced physical activity (X^2^ = 6.6, *p* = 0.010), suggesting these to be the key drivers.

For those living with a pre-existing long-term condition affecting the foot, the impact of the COVID lockdown appeared to amplify physical restriction. For example, one respondent with rheumatoid arthritis wrote:

*“It’s harder to get the joints moving and my toes are a bit swollen. The process of gardening, bending at the ankles, balancing etc. is causing more foot pain … people who have been shielding have been far less mobile than usual and we need to get back on track again, ASAP!” (participant #11).*

The opportunity for enthusiastic increased activity in early lockdown resulted in new injury for some:

*“I wish I’d taken running easier to start with – ‘going for a run’ seems so ubiquitous and easy … but if out of condition it’s not!” (participant #55).*

Amongst people with a new foot problem, the disruption to support networks caused by COVID lockdown was also reported; having sustained an acute foot injury there was notable frustration at the lack of support or guidance available to aid recovery. Thus, the importance of support needed to recover or maintain foot health does not appear to be weighted toward either acute or long-term conditions and instead is common across both groups.

The reasons given for foot health restricting physical activity were thematically grouped into physical or emotive restrictions. Reported physical restrictions included reduced ability to walk a certain distance, being slower, moving or standing differently, or sensations of tightness, weakness, or pain.

Reported emotive restrictions included fear of movement or lacking motivation.

## Discussion

This study has measured the impact of COVID-19 restrictions upon foot health, demonstrating an association between poor foot health or foot pain and reduced physical activity during lockdown. Importantly, the findings show how the pandemic restrictions have interrupted support networks integral to maintaining foot health. Specifically, the lockdown disrupted relied-upon support networks, both formal, e.g., healthcare providers, and informal, e.g., friends or family members. The over-arching theme of disrupted support networks was informed by five sub-themes: 1. foot pain is a constant companion, 2. self-care, 3. ‘cope or crumble’ scenarios, 4. future intent to access healthcare and 5. reduced ability to undertake physical activity. Themes were inter-related, for example, foot pain was related to changes in physical activity and people’s perceived inability to self-care, which in turn related to future intent to access health professional support. Perceived ability to self-care and disruption to support networks forced the emergence of a ‘cope or crumble scenario’ and, in a sometimes-cyclical fashion, continued poor foot health impacted people’s ability to remain physically active, leading to further increased pain or generally poorer health.

The findings are consistent with those of other authors, who have reported detrimental impact to physical activity due to lockdown [2, 4, 5, 8, 21]. However, we have demonstrated that foot health is also a key contributing factor to being able to undertake physical activity during lockdown. This work reinforces the findings of previous work [17], noting that there is a need to consider the impact of foot health in terms of daily physical activity and more broadly, maintaining general physical health or recovering from illness [12, 22].

Our findings demonstrate that foot problems previously thought of as relatively ‘minor’ by respondents, such as support for skin and nail care, were exacerbated by restricted support networks leading to greater detrimental impact upon health and wellbeing. Thus, the implication of this work for those coordinating policy or public health response, is to consider prioritisation of the integrated breadth of service provision rather than full restriction to services not directly associated with acute care; however short-term such restrictions may seem, our findings suggest that after 1–2 months, difficulties were already manifest and with the implications of resultant escalating medical complexity and reduced physical activity [11, 23, 24]. Similarly, the emotive impact of perceived poor foot health or complex pain related beliefs and behaviour is worthy of further consideration. There is possibly an unmet need relating to facilitation of person centred care that has not been well supported during lockdown. Whilst physical interventions may be restricted, learning from other disciplines and application to virtual foot health consultation could provide useful facilitation of supported self-management [18, 25].

Most respondents were attempting to engage with some form of self-care. However, on-the whole, self-care alone was not considered sufficient to alleviate the problems. Thus, our findings suggest that greater resource is needed to support self-care and to prepare the general population to undertake changes in physical activity that could be associated with mandated lockdown. It is also noteworthy that despite reported high levels of self-care, people remained intent on re-accessing health care services for support as soon as they were able to do so (theme 4). This could lead to capacity challenges once services are made available and providers may need to prepare for the sudden increase in demand, however there is limited evidence to date about the impact of service access post-lockdown restriction particularly relating to allied health care services. To mitigate this possibility, there appears to be opportunity to increase support for self-care by enabling support networks, which are integral to maintaining foot health. There is opportunity to integrate a persons support network with professional-led services to enable improved foot health; exploration of collaborative opportunities for private enterprise, voluntary or charitable sectors, and nationally funded health services to work in a harmonised fashion could bring greater benefit, health care equity, and service efficiencies.

Similarly, a lack of previously published work suggests that foot health could receive greater public health focus and clearer public messaging about physical activity and foot health [26]. However, the rapid uptake of technology during the pandemic combined with the notable intent to self-care demonstrated in our study, arguably provides opportunity for innovation in public health and education messaging [27]. Further research is required to develop evidence that can underpin public health messages associated with foot health and show their efficacy at improving levels of physical activity.

There are limitations to this work that should be considered. Due to the nature of the sampling strategy employed, it was not possible to determine the extent of response bias evident within the sample. Therefore, the data reported here cannot be considered in any way indicative of estimates of prevalence or incidence for poor foot health in the general population. None the less, following qualitative data collection, included via the open survey questions, the thematic analysis align with the priority weighted quantitative results. It should however be considered that Creswell et al. acknowledge the potential limitation of applying qualitative methods within the mixed methodological framework of survey conduct with quantitative weighting, noting the potential for limited depth of understanding or context. It is also noteworthy that there is a gender imbalance in respondents, with more females than males contributing to the survey results. The underlying reason for this is unclear however Lopez-Lopez et al. [17] also noted gender differences foot-related quality of life. Similarly, the inclusion criteria for the study did not restrict international participation, it is noteworthy that most respondents were experiencing COVID-19 lockdown from within the UK. As such, the generalisability of the results could be considered mostly applicable to the UK setting only.

## Conclusions

Pain was the most frequently reported foot problem during COVID-19 lockdown restriction. Lockdown restrictions disrupted support networks integral to maintaining foot health. Poor foot health impacted people’s ability to remain physically active. Complaints previously considered relatively ‘minor’ such as support for skin and nail care, were found to be exacerbated by restricted support networks, leading to greater negative impact.

## Supplementary Information

**Additional file 1.** Qualitative themes, subthemes, and codes.

## Data Availability

The dataset used and analysed during this study is available from the corresponding author on reasonable request.
